# Work from home today for a better tomorrow! How working from home influences work‐family conflict and employees' start of the next workday

**DOI:** 10.1002/smi.3053

**Published:** 2021-05-06

**Authors:** Maral Darouei, Helen Pluut

**Affiliations:** ^1^ Department of Management and Organisation School of Business and Economics, Vrije Universiteit Amsterdam Amsterdam The Netherlands; ^2^ Department of Business Studies Leiden Law School, Leiden University Leiden The Netherlands

**Keywords:** daily diary study, time pressure, well‐being, work‐family conflict, working from home

## Abstract

Drawing on the resource (drain) perspective in work‐family spillover theory and conservation of resources theory, the current paper studies the daily consequences of working from home for employees' work‐home interface and well‐being. We build an intraindividual model that investigates how working from home influences experiences of time pressure, work‐family conflict, and work‐related employee well‐being on a daily basis. A total of 34 professional workers participated in our study and were asked to respond to 10 daily surveys in the morning, afternoon and evening, across two consecutive workweeks. In line with our hypotheses, results indicated that on days when employees worked from home, they experienced less time pressure and, in turn, they reported lower levels of work‐family conflict on that particular day. Moreover, we found that experiences of work‐family conflict predicted individuals' next morning engagement and exhaustion levels and affective states towards the organization they work for. We recommend organizations to encourage a work‐from‐home protocol aimed at protecting employee well‐being.

## 1 INTRODUCTION

Today, 32% of employees in the EU struggle to fulfil family responsibilities because of pressing job demands (Eurofound, 2018). Striking a balance between work and family is crucial as it has a significant impact on employees' well‐being (OECD, 2017). Given the commonality of today's high‐pressure work environments (Prem et al., 2018), concerns are being raised about how employees can overcome the detrimental effects of high job demands and achieve a satisfactory work‐life balance (De Hauw & Greenhaus, 2015). These concerns have urged organizations to re‐evaluate their employment policies and seek alternative forms of working, such as telecommuting. Telecommuting, often referred to as telework or working from home, is a policy that enables employees to perform their job at home during some part of the week and stay connected to the office by means of communication technologies (Allen et al., 2015). Precipitated by the COVID‐19 pandemic, an increasing number of firms have implemented telecommuting arrangements, with the hope that employees can better manage their work‐home interface and safeguard their well‐being (Kelliher & de Menezes, 2019; Matos et al., 2016). Yet, is it effective?

Interest in the effectiveness of the working from home practice for employees' work‐home interface and well‐being is reflected in the academic literature, with a growing body of research on the effects of telework on work‐family conflict (Delanoeije et al., 2019; Schieman & Young, 2010). Work‐family conflict is ‘a form of interrole conflict in which the role pressures from the work and family domains are mutually incompatible in some respect’ (Greenhaus & Beutell, 1985, p. 77). Numerous studies have shown a negative association between telework and work‐family conflict (see Allen et al., 2013, for a meta‐analysis), indicating that the practice can be used as a means to alleviate conflict between the two life domains. The vast majority of such studies, however, have taken a between‐individual approach, where work‐family conflict experiences of teleworkers are compared with those of full‐time office workers (Allen et al., 2015).

This approach may limit our understanding of the consequences of working from home in at least two ways. First, the teleworker versus full‐time office worker perspective does not portray a realistic picture of how the working from home policy is used in practice. In fact, individuals rarely work from home every day but rather combine working from home days with office days (Biron & Van Veldhoven, 2016). This hybrid approach is expected to increase in the wake of change from the COVID‐19 pandemic, as many businesses are splitting staff into teams with alternating working from home days to ensure social distancing (Marin‐Guzman, 2020). Examining day‐specific effects of working from home matches the increasing number of employees who alternate between office and working from home days, thereby enhancing our understanding of how the practice is currently being used.

Second, previous cross‐sectional (i.e., between‐person) research has been overly focused on the implications of employees' perceptions of the availability of telework arrangements (Erden & Bayazit, 2019; Masuda et al., 2012). Scholars lack a thorough understanding of what happens on days that employees utilize the practice (Delanoeije & Verbruggen, 2020). As a consequence, organizations and employees run the risk that telework arrangements are adopted, used, and managed without a proper understanding of its effects and effectiveness. Conceptualizing working from home at the intraindividual level and studying day‐to‐day fluctuations in working from home can broaden our theoretical and practical insights into the relatively short‐term consequences of the utilization of the telework arrangement.

There are a few recently conducted studies on working from home that help us understand the effectiveness of workplace flexibility as it is currently being employed and used. Taking a resource perspective, Delanoeije and Verbruggen (2020) examined the daily relationships between working from home and four outcomes that have been frequently studied in relation to working from home, namely work‐family conflict, work engagement, stress, and performance (for a meta‐analysis, see Allen et al., 2015). They found that employees reported less work‐family conflict and stress and experienced higher work engagement and performance on working from home days. Similarly, Vega, Anderson, and Kaplan (2015) used diary data to investigate the daily relationship between working from home and job‐related affective well‐being and found that employees experienced less negative affective well‐being and more positive affective well‐being on days when they worked from home.

**FIGURE 1:**
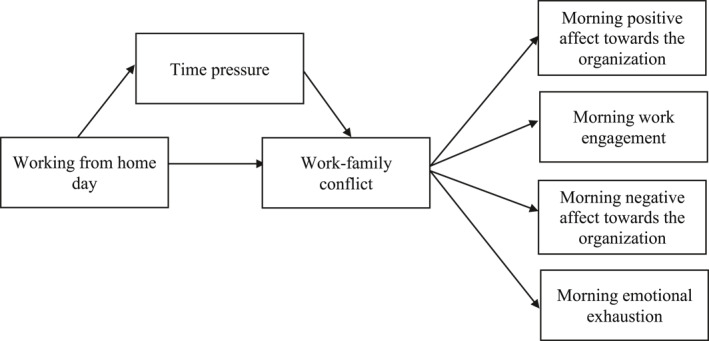
Conceptual model

While these studies have broadened our sight on the daily relationships between working from home and work‐family conflict on the one hand and work‐related well‐being on the other hand, it remains elusive how these concepts relate to each other and what are the mediating pathways. We therefore build an intraindividual model that integrates these key concepts and examines their interplay in order to elucidate the daily effects of working from home. As research has only just begun to examine the relationship between telework and work‐family conflict on a daily level (e.g., Delanoeije et al., 2019), we aim to contribute to this stream of research by examining to what extent experiences of work‐family conflict fluctuate across office versus working from home days. We go beyond prior research on working from home in at least two noteworthy ways. First, while previous research has focused on general stress as an outcome of daily working from home (e.g., Delanoeije & Verbruggen, 2020), we propose that time pressure is an important mechanism (i.e., mediator) that explains the daily relationship between working from home and work‐family conflict. Second, to the best of our knowledge, no study has yet investigated cross‐day effects of work‐family conflict and we consider this an important gap to fill. By examining how work‐family conflict influences the psychological states of employees the next day, our research may help organizations to better understand the implications of working from home for the upcoming workday. Specifically, we focus on how work‐family conflict experiences in the evening relate to employees' work engagement, emotional exhaustion, and affect towards the organization the next morning. Our full conceptual model is presented in Figure 1.

## 2 THEORETICAL DEVELOPMENT OF THE CURRENT STUDY

### 2.1 Daily consequences of working from home

In building our conceptual model, we draw on the resource (drain) perspective in work‐family spillover theory (Edwards & Rothbard, 2000). Resources, such as time, attention and energy, are finite and once expended in one domain become unavailable for other domains (Eckenrode & Gore, 1990). We focus on work interfering with family^1^ as an outcome related to the work‐home interface, as previous studies have shown that working from home affects work‐to‐home conflict more so than home‐to‐work conflict (Allen et al., 2015; Delanoeije et al., 2019). On a demanding workday, employees' personal resources are more likely to be drained, leaving them with fewer resources in the family domain (Ten Brummelhuis & Bakker, 2012). Time‐based and strain‐based work‐family conflicts refer to situations in which work consumes time and energy, respectively, that cannot be spent at home (Greenhaus & Beutell, 1985). Interestingly, individuals' work‐family conflict experiences are likely to vary daily as a result of day‐to‐day fluctuations in job demands (Ilies, Schwind, Wagner, et al., 2007; Pluut et al., 2018). Employees' daily work environment may be an important antecedent of daily variations in job demands. In fact, past research has found that telework may influence the extent to which individuals experience job demands (Peters & Van der Lippe, 2007). Following this logic, employees' daily experiences of work‐family conflict may be influenced strongly by whether they work at the office or from home on a given day.

Yet, studies on the consequences of telework for work‐family conflict have primarily taken a between‐person approach (for exceptions, see Delanoeije et al., 2019; Delanoeije & Verbruggen, 2020). Based on the above and considering that employees' use of the telework practice is volatile (i.e., employees rarely work from home every day), we conceptualize telework at the intraindividual level and examine its effects on work‐family conflict on a daily basis. Here we propose time pressure as a mediator because time is a scarce personal resource for employees that they might find easier to protect in a work environment that allows for a more fungible use of time (Borpujari et al., 2020). Employees' daily work environment (i.e., home or office) may impact the drain of this resource such that time pressure varies across days. Time pressure is a commonly experienced job demand that has been found to cause work‐family conflict (Brosch & Binneweis, 2018; De Carlo et al., 2019). Hence, we take a resource (drain) perspective and examine how working from home is related to a key precursor of work‐family conflict, namely time pressure.

### 2.2 Cross‐day effects of work‐family conflict

We further use the resource loss spiral principle of conservation of resources (COR) theory (Hobfoll, 1989; Hobfoll et al., 2018) to propose that the resource drain associated with work‐family conflict may extend to the next workday. In particular, we integrate COR theory with the resource (drain) perspective in work‐family spillover theory (Edwards & Rothbard, 2000) to extend our understanding of what happens when work and family interfere (i.e., consequences of work‐family conflict). COR theory posits that once resources are lost, individuals become more vulnerable to further resource loss and may find themselves in a resource loss spiral. Researchers have examined the long‐lasting impact of resource loss as well as the role that resources play on the shorter term, such as across days or weeks (Airila et al., 2014; Demerouti et al., 2015; Donald et al., 2016). We propose that work‐family conflict (which refers to a situation in which resources are depleted) influences how employees feel about their upcoming workday. Specifically, we examine how experiences of work‐family conflict in the evening influence work‐related well‐being the next morning. In our examination of work‐related well‐being, we follow a recent line of research that integrates positive and negative perspectives on well‐being in the workplace (Van den Tooren & Rutte, 2016) by focussing on work engagement, emotional exhaustion, and positive and negative affect towards the organization.

### 2.3 Hypotheses

Individuals experience work‐family conflict when demands from work deplete personal resources (e.g., time and energy) and consequently hamper performance at home (Edwards & Rothbard, 2000; Ten Brummelhuis & Bakker, 2012). On days when employees work from home instead of at the office, they may find execution of their work role less demanding. In fact, a vast body of research has shown that telecommuting is negatively related to work role stress (Allen et al., 2015; Delanoeije & Verbruggen, 2020; Gajendran & Harrison, 2007) and work exhaustion (Sardeshmukh et al., 2012). In line with work‐family spillover theory, this would imply that working from home reduces the likelihood of experiencing negative spillover from work to family because employees are left with more resources that can be used to actively participate in the family role. While working from home may blur the boundaries between work and family (Pluut & Wonders, 2020) and hence result in work‐family conflict (Schieman & Young, 2010), from a resource (drain) perspective, it should reduce work‐family conflict. Indeed, the majority of studies on the relationship between telework and work‐family conflict show a negative association between the two constructs (see Allen et al., 2013, and Gajendran & Harrison, 2007, for meta‐analyses). Although most research on the association between telework and work‐family conflict has employed a between‐individual approach, recent intraindividual research supports our claim by showing that on teleworking days individuals experience less work‐to‐home conflict than on office days (Delanoeije et al., 2019). We aim to replicate this finding and hypothesize:

Hypothesis 1 *Within individuals, working from home (compared with at the office) will be negatively associated with work‐family conflict*.

Next, we expect that time pressure elucidates the negative relationship between telework and work‐family conflict. Time pressure is a work‐related stressor that refers to the experience of having to work at a fast pace or having insufficient time to complete work‐related tasks (Baer & Oldham, 2006). We argue that on days when employees work at home, they experience less time pressure. There are several reasons to expect such an effect. First, on working from home days, employees have significantly reduced contact with their colleagues and supervisors that may keep them from focussing on their work duties (Windeler et al., 2017). Fewer work‐related interruptions and distractions may enhance individuals' concentration levels and help employees in completing more (complex) tasks (see Smit et al., 2016). This increased productivity may reduce employees' sense of urgency and feelings of having to speed up the work pace. A second explanation for why employees may experience less time pressure on a working from home day is the greater autonomy in deciding how and when to perform tasks (Gajendran et al., 2014). Control over scheduling one's own working day can be used to schedule work in an efficient manner, thus saving energy and time. Finally, working from home may have a time pressure‐reducing potential because it eliminates commuting time and effort (Peters et al., 2004), leaving the employee with more time for work duties. When employees know they have more time to allocate to work, they are less likely to feel anxious and under pressure about the work‐related tasks they have to complete that day.

Lending support to the above arguments, research suggests that working from home reduces experiences of time pressure (Peters & Van der Lippe, 2007). In their cross‐sectional study among 807 employees in the Netherlands, Peters and Van der Lippe (2007) showed that employees working from home more than one day per week on average experience less time pressure than full‐time on‐site workers. Thus, we expect that on working from home days, individuals experience less time pressure than on office days. Time pressure, in turn, may be a strong predictor of daily work‐family conflict. Dealing with time pressure on a given day may keep individuals from actively participating in the family role because of depleted (emotional) resources (Pluut et al., 2018; Prem et al., 2018). In line with the resource (drain) perspective in work‐family spillover theory, we put forward the following hypothesis:

Hypothesis 2 *Within individuals, time pressure mediates the negative relationship between working from home and work‐family conflict experienced at home*.

On days when employees are not able to satisfy the needs of the home domain due to the demands of the work role (i.e., work‐family conflict), they may experience stress because they could not successfully manage both roles (Grandey & Cropanzano, 1999). We posit that work‐family conflict is an exhausting and resource‐draining experience for two reasons. First, past research has suggested that experiences of work‐family conflict may lead to a negative state of being, including negative emotions such as anxiety and dissatisfaction (Greenhaus et al., 2006). Judge and colleagues (2006), for instance, showed that on days when employees' work interferes with the family role, they experience more negative emotional responses (i.e., hostility and guilt) at home. Second, when stress arises from the incompatibility of two salient life roles, the individual is likely to ruminate about ‘whether and how one can fix the issues causing the conflict and the potential consequences of the conflict’ (Davis et al., 2016, p. 330). In order to overcome negative emotions and prevent becoming stuck in rumination, the individual is likely to engage in self‐regulation (Muraven & Baumeister, 2000) and employ personal resources (e.g., optimism) that he or she possesses (Liu et al., 2015), to offset further resource loss. Put differently, an individual who experiences work‐family conflict may decide to expend additional mental effort to think optimistically and alter their naturally occurring negative emotions.

In line with the resource loss principle of COR theory (Hobfoll et al., 2018), then, conflict between work and family may negatively affect well‐being the next morning. We know from past empirical work that work‐related well‐being has a state‐like component and fluctuates on a daily basis (Pluut et al., 2018; Sonnentag et al., 2012). Day‐level variations in well‐being constructs can be explained by fluctuations in personal resources (Liu et al., 2015). As we argue that individuals who experience work‐family conflict are more likely to start the next morning with inadequate personal resources, we expect that day‐level variations in work‐family conflict explain fluctuations in employees' levels of emotional exhaustion and work engagement the next workday.

First, several studies have shown that work‐family conflict is positively associated with burnout and emotional exhaustion (for a review, see Allen, Herst, Bruck, & Sutton, 2000). Taking a resource drain perspective, Simbula (2010), for instance, showed that at the within‐person level, work‐family conflict experiences predict emotional exhaustion. Moreover, there is empirical evidence for the longitudinal effects of work‐family conflict on emotional exhaustion and burnout (Hall et al., 2010; Leineweber et al., 2014). Although previous research has shown that work‐family conflict predicts emotional exhaustion on the day level and the long term, we know little about how daily work‐family conflict experiences influence the next day, specifically how employees feel about the upcoming workday. Based on the above theoretical arguments and empirical insights, we put forward the following hypothesis:

Hypothesis 3 *Within individuals, work‐family conflict experienced at home in the evening is positively related to emotional exhaustion the next morning*.

Second, we expect that the effect of daily work‐family conflict on next morning work‐related well‐being is not limited to feelings of exhaustion but that it also affects engagement. Employees who have enough personal resources (e.g., high levels of energy) are likely to be engaged in their work. Research has indeed demonstrated that feeling recovered and refreshed in the morning (i.e., having energetic resources) helps employees to feel engaged in their work during the day (Kühnel et al., 2012; Lanaj et al., 2014). When employees find themselves in a resource‐depleting situation (i.e., work‐family conflict), however, they may decrease their level of engagement to protect their remaining resources (Babic et al., 2017). Cross‐sectional studies have indeed shown that work‐family conflict is negatively associated with engagement (Opie & Henn, 2013), and this negative association (with vigor in particular) appears to hold over time (see Mauno et al., 2007, for a 2‐year study). It remains to be studied, however, how work‐family conflict and engagement relate across days. Based on the above empirical insights and in line with the resource loss principle of COR theory, we expect that experiences of work‐family conflict in the evening reduce individuals' work engagement the next morning.

Hypothesis 4 *Within individuals, work‐family conflict experienced at home in the evening is negatively related to work engagement the next morning*.

So far, we have proposed that experiences of work‐family conflict deplete personal resources and leave employees with scarce energy to start the next workday. We further argue that work‐family conflict influences individuals' affect towards the organization. Failing to meet family demands because of work is unpleasant and thus may trigger negative affective reactions (Livingston & Judge, 2008). Studies using within‐individual designs have indeed found that work‐family conflict predicts negative emotions, such as guilt and hostility (Judge et al., 2006). Importantly, when work and family interfere, employees seek a cause for their negative emotions (Ilies et al., 2012). The source attribution perspective of work‐family conflict (Shockley & Singla, 2011) entails that employees are likely to psychologically attribute blame to the source of the conflict and become dissatisfied with that role (see also Zhao et al., 2019, and Speights et al., 2019). In the case of work interfering with family, it means that individuals may perceive the organization they work for as the cause (because work is the source of conflict) and thus attribute their negative emotions to their organization. This perspective is supported by past cross‐sectional research showing that when work interferes with family, individuals appraise their work negatively, become dissatisfied with their job, and show less commitment to their organization (see Amstad et al., 2011, for a meta‐analysis). Integrating previous within‐individual research that has shown that state‐level emotions can last until the next day (Tremmel & Sonnentag, 2018) with the source attribution perspective of work‐family conflict (Shockley & Singla, 2011), we hypothesize that experiences of work‐family conflict in the evening increase feelings of negative affect and reduce feelings of positive affect towards the organization the next morning.

Hypothesis 5a *Within individuals, work‐family conflict experienced at home in the evening is positively related to negative affect towards the organization the next morning*.

Hypothesis 5b *Within individuals, work‐family conflict experienced at home in the evening is negatively related to positive affect towards the organization the next morning*.

In sum, we propose that on days when employees work from home, they are less likely to experience work‐family conflict than on office days, and this relationship is explained by reduced time pressure. Moreover, we propose that the effects of work‐family conflict spill over to the next workday, in terms of employees' exhaustion and engagement levels in the morning and how they feel (i.e., positive and negative affect) about the organization they work for.

## 3 METHOD

### 3.1 Sample and procedure

We posted an online application form on network platforms, such as LinkedIn, to recruit professional workers in the Netherlands. In order to qualify for participation in the study, the individual needed to work from home at least two days a week and live together with others in the household. Furthermore, the invitation indicated a preference for partner participation. A total of 34 individuals and 24 partners indicated to be eligible and agreed to participate in our daily research study. As an appreciation for participants' effort, 10 raffle prizes were distributed among the participants. Winners were randomly selected from all eligible participants. Prior to the start of the diary study, participants were requested to fill out a one‐time online questionnaire, which assessed demographic variables. All of the respondents completed the initial survey. The majority of the participants indicated to be in a relationship (82%) and a total of nine respondents (27%) indicated to have children. The vast majority of the sample consisted of women (68%). The age of respondents ranged from 25 to 58, with a mean of 33 years. On average, participants worked 38 hours and worked from home 2.7 days a week. Individuals held jobs in various sectors, such as the legal sector, academia, and IT.

During two consecutive workweeks, individuals were required to fill out three daily web‐based surveys, one in the morning at home, one in the afternoon while working (either at home or at the office) and one in the evening at home. Participants were instructed to answer the morning questions within an hour of waking up, fill out the afternoon questionnaire within an hour of finishing work, and respond to the evening surveys within an hour of going to bed. During this same period, the spouse of the participant received one survey each evening and was asked to fill out the survey before going to sleep. In order to protect the anonymity of each individual, participants were requested to create an identification code, which was used to link their records across days. Spouses were asked to use the same code as their partner, which we could then use to link the answers of participants and their spouses. Given that the recorded surveys contained a time stamp, we could check whether respondents filled them out on the same day. Evening surveys that were completed the day after were removed for further analyses. Our final sample consists of 34 participants, who provided 324 daily records with an average of 9.4 days per person out of a maximum of 10 days. In terms of the spouse sample, we obtained 205 out of a possible 240 daily responses from 24 participants, with an average of 8.5 days per person.

### 3.2 Measures

**Workplace.** As part of the morning survey, respondents were asked to indicate whether they would work from home or at the office on that particular day. We then assigned a code to each category, where 0 indicates an office day and 1 represents a working from home day.

**Time pressure.** Employees' daily experience of time pressure was measured in the afternoon survey. We used three items that concerned specifically time pressure from the five‐item workload scale previously used by Pluut and colleagues (2018). We asked respondents to indicate their agreement with the following three statements: ‘I worked under time pressure today’, ‘Today, I had to work fast’, and ‘I had problems with the pace of work today’ on a five‐point Likert scale ranging from 1 = *strongly disagree* to 5 = *strongly agree*. Across days, the average α was 0.82.

**Work‐family conflict.** Work‐family conflict was assessed using the five‐item work‐family conflict scale developed by Netemeyer et al. (1996). Following other intraindividual studies who used this scale (e.g., Derks et al., 2016), we slightly modified the items to capture employees' daily work‐family conflict experiences. Each evening, within an hour of going to bed, respondents rated the level of experienced work‐family conflict with statements such as ‘Today, my job produced strain that made it difficult to fulfil family duties’. Responses were recorded using a five‐point Likert scale ranging from 1 = *strongly disagree* to 5 = *strongly agree*. The average α across evenings was 0.93.

In the spouse survey, we assessed the perceptions of partners regarding the level of work‐family conflict of the focal participants. We used the same items as for the self‐reports of work‐family conflict but changed the referent. For instance, the above item was altered into ‘Today, my partner's job produced strain that made it difficult for him/her to fulfil family duties’. Each evening, spouses were asked to indicate their agreement with the statements on a five‐point Likert scale ranging from 1 = *strongly disagree* to 5 = *strongly agree*. Across evenings, the average α was 0.94.

**Work engagement.** Employees' daily engagement was measured in the morning within an hour of their wake‐up time with the Utrecht Work Engagement Scale (UWES; Schaufeli et al., 2006). The nine‐item UWES consists of vigor, absorption, and dedication as dimensions of engagement. To measure state work engagement, scholars have created an adapted version of the UWES, which has been validated using daily diary data (Breevaart et al., 2012). We slightly modified Breevaart and colleagues' items to measure work engagement in the morning instead of retrospectively in the evening. Moreover, given that the absorption dimension of the UWES is only relevant at the end of the workday, we excluded it from our scale. We asked respondents to indicate their agreement with statements such as ‘This morning, I feel strong and vigorous when I think about my job’ (vigor) and ‘This morning, I am enthusiastic about my job’ (dedication) on a five‐point Likert scale ranging from 1 = *strongly disagree* to 5 = *strongly agree*. Our six‐item measure of daily engagement had an average Cronbach's alpha of 0.91 across days.

**Emotional exhaustion.** We measured emotional exhaustion in the morning survey with six items from the emotional exhaustion subscale of the Maslach Burnout Inventory (Maslach & Jackson, 1981). The items were slightly altered to capture individuals' daily experiences of emotional exhaustion. Each morning, within an hour of waking up, participants were requested to respond to items such as ‘When I got up this morning, I felt too fatigued to face another day on the job’. Answers were recorded on a five‐point Likert scale ranging from 1 = *strongly disagree* to 5 = *strongly agree*. Across days, the average α was 0.91.

**Positive and negative affect towards the organization.** Following previous studies on affect towards the organization (see e.g., Caesens et al., 2016), we measured affective states towards the organization using the Positive and Negative Affect Schedule (PANAS; Watson et al., 1988). As in Panaccio and Vandenberghe's (2009) study, we used the introductory sentence ‘Right now, when I think about the organization I work for, I feel….’, followed by several positive (e.g., interested, excited) and negative (e.g., jittery, distressed) adjective descriptors. We used a total of 10 descriptors from a shortened version of PANAS that were administered to respondents in the morning. Responses were recorded on a five‐point Likert scale ranging from 1 = *very slightly or not at all* to 5 = *extremely much*. Positive affect (PA) and negative affect (NA) had an average α of 0.94 and 0.69 across mornings, respectively.

### 3.3 Analyses

The data used for the analyses have a nested structure, where days (Level 1; *n* = 324) are nested within individuals (Level 2; *n* = 34). Before conducting the analyses, we calculated the between‐individual and within‐individual variance components of our study variables, by estimating null models (i.e., no predictors) for each construct. The percentage of variance due to within‐individual variation in construct scores ranged from 18% (morning organizational PA) to 89% for the workplace variable (see Table 1). The overall high day‐to‐day fluctuations of our study variables confirm that within‐individual analyses are suitable to test our model.

**TABLE 1:** Variance components of null models for Level‐1 variables

Study variable	Within‐individual variance (*σ* ^2^)	Between‐individual variance (*τ* ^2^)	Percent variability within individuals
Workplace	0.225	0.026	89.5
Time pressure	0.786	0.354	69.0
Work‐family conflict (employee‐rated)	0.804	0.346	69.9
Work‐family conflict (spouse‐rated)	0.600	0.404	59.8
Morning work engagement	0.313	0.488	39.1
Morning organizational PA	0.187	0.826	18.4
Morning emotional exhaustion	0.334	0.456	42.3
Morning organizational NA	0.081	0.129	38.6

*Note:* *N* = 34. Percent variability within individuals was computed as *σ* ^2^/(*σ* ^2^ + *τ* ^2^)*100.

Abbreviations: NA, negative affect; PA, positive affect.

All within‐individual variances were significantly different from zero (*p* < 0.001).

We used hierarchical linear modelling (HLM 6; Bryk & Raudenbush, 1992) to test our theoretical model. Each level‐1 predictor variable was centred relative to the individuals' means across days on the focal variables. In this way, the scores signify deviations from an individual's respective mean, and ‘the subject serves as his or her own control’ (DeLongiset al., 1988, p. 487).

## 4 RESULTS

The descriptive statistics and correlations are presented in Table 2.

**TABLE 2:** Within‐individual and between‐individuals correlations of study variables

	*M*	*SD*	1.	2.	3.	4	5.	6.	7.	8.
1. Workplace^a^	0.48	0.23		−0.29***	−0.31***	−0.21	0.02	0.01	−0.06	0.01
2. Time pressure	2.78	0.66	−0.29		0.26**	0.19	−0.08	0.03	0.25	0.16
3. Work‐family conflict (employee‐rated)	2.07	0.67	−0.23	0.37*		0.40**	−0.19**	−0.07	0.34**	0.20**
4. Work‐family conflict (spouse‐rated)	1.86	0.98	−0.34	0.26	0.50*		−0.01	−0.04	0.27*	0.16*
5. Morning work engagement	3.19	0.73	−0.13	0.12	−0.15	0.15		0.38***	−0.50***	−0.05
6. Morning organizational PA	2.62	0.92	0.02	0.12	−0.08	0.25	0.78**		−0.11	0.15
7. Morning emotional exhaustion	1.88	0.70	0.07	0.12	0.35*	0.13	−0.73**	−0.62**		0.29***
8. Morning organizational NA	1.35	0.37	−0.06	0.23	0.11	0.15	−0.17	−0.02	0.41*	

*Note:* Means (*M*) and standard deviations (SD) are between‐individual descriptive statistics. The correlations below the diagonal represent between‐individual associations, which are calculated based on individuals' aggregated scores (*n*s = 34 to 24, pairwise). The correlations above the diagonal represent within‐individual associations and are calculated using the group‐mean centred scores (*n*s = 230 to 302 for correlations involving self‐reported scores and *n*s = 152 to 192 for spousal ratings).

Abbreviations: NA, negative affect; PA, positive affect.

*p < 0.05; **p < 0.01; ***p < 0.001

^a^ Workplace: working at the office = 0, working from home = 1.

As a first step, to test Hypothesis 1, we regressed work‐family conflict on workplace. Lending support to our first hypothesis, the results showed that on days when employees worked from home, they experienced less work‐family conflict compared with days on which they worked at the office (*B* = −0.60, *p* < 0.001). We then used the procedures of Bauer and colleagues (2006) to holistically test a model in which time pressure mediates the path between workplace and work‐family conflict. In support of Hypothesis 2, the findings indicated that working from home was negatively associated with time pressure (*B* = −0.55, *p* < 0.001) and time pressure was positively related with work‐family conflict (*B* = 0.25, *p* = 0.002). Thus, both paths of the mediation model were significantly different from zero. As a next step, we employed an R package called ‘RMediation’ (Tofighi & MacKinnon, 2011) to test our mediation hypothesis directly. This package produces indirect effect estimates and generates confidence intervals around the effects on the basis of the distribution‐of‐the‐product method. RMediation estimated the indirect effect of workplace to work‐family conflict via time pressure at −0.14 with a 95% CI of [−0.251, −0.049]. On days when employees worked from home, they felt less time pressure and consequently experienced less work‐family conflict, compared with office days. These results provide support for Hypothesis 2.

To test our third and fourth hypothesis, we regressed emotional exhaustion and engagement on work‐family conflict, respectively. We observed that on evenings when individuals experienced heightened levels of work‐family conflict, they felt more emotionally exhausted (*B* = 0.20, *p* = 0.004) and less engaged (*B* = −0.12, *p* = 0.010) the next morning. Finally, we regressed positive and negative affect towards the organization on work‐family conflict, to examine Hypothesis 5. Lending support to Hypothesis 5a, the findings indicated that on days when individuals experienced more work‐family conflict, they felt more negative emotions towards the organization the upcoming workday (*B* = 0.06, *p* = 0.007). Within individuals, experiences of work‐family conflict did not predict positive affect towards the organization the next morning (*B* = −0.03, *p* = 0.588), which leads us to reject Hypothesis 5b.^2^

### 4.1 Additional analyses

To reduce the common rater day‐specific bias concern related to experience sampling methodology (Ilies, Schwindt, & Heller, 2007), we replicated our mediation analyses with spouse‐rated work‐family conflict as an outcome. Using spousal ratings, we did not find support for Hypothesis 2, which states that time pressure mediates the negative relationship between workplace and work‐family conflict (indirect effect = −0.053, 95% CI of [−0.145, 0.026]). However, the direct effect of workplace on spouse‐rated work‐family conflict was significant (*B* = −0.36, *p* = 0.007). In other words, spouses confirmed that on days when employees worked from home, work was less likely to interfere with the family domain. We also replicated our cross‐day analyses with spouse‐rated work‐family conflict as a predictor. The findings were in line with the results found for the cross‐day effects of employee‐rated work‐family conflict. That is, in support of H3, H4 and H5a, we found that spouse‐rated work‐family conflict predicted emotional exhaustion (*B* = 0.17, *p* = 0.009), work engagement (*B* = −0.14, *p* = 0.044), and negative affect towards the organization the next morning (*B* = 0.06, *p* = 0.013). Moreover, in line with our previous finding for Hypothesis 5b, the effect of spouse‐rated work‐family conflict on positive affect towards the organization the next morning was not significant (*B* = −0.03, *p* = 0.721).

## 5 DISCUSSION

Our intraindividual study aimed to elucidate the process by which working from home affects employees' work‐home interface and consequently work‐related well‐being. Integrating work‐family spillover theory with the resource loss spiral principle from COR theory, we argued that on days when employees work at the office, they are more likely to (a) lose resources and (b) find themselves in a loss spiral. In line with the first argument, we demonstrated that on office days, individuals experienced more work‐family conflict, through greater perceptions of time pressure. In addition, we found that on working from home days employees reported lower levels of work‐family conflict, which was confirmed by spouse reports. Yet, we did not find support for the mediating effect of time pressure on the relationship between workplace and spouse‐rated work‐family conflict. It may be that time pressure as a work stressor is less noticed by the partner. This finding is in line with research that posits that some work‐related demands are less observable by the spouse and may be perceived by partners as less interfering with family participation (Ilies et al., 2015).

Lending support to the second argument, which posits that employees find themselves in a loss spiral when they experience work‐family conflict, we illustrated that employees start the next morning feeling emotionally exhausted and less engaged and they have higher negative affect towards the organization when work has interfered with the family domain the previous day. Interestingly, experiences of work‐family conflict in the evening did not predict employees' positive affect towards the organization the next day. An explanation for this finding might be that work‐family conflict is a negative situation, and positive affective states correspond with positive events instead of negative events (Gable et al., 2000). It should also be noted that positive affect showed very low within‐person variability (see Table 1; 18%), suggesting that it is less sensitive to daily fluctuations.

### 5.1 Strengths and implications for research

Our findings contribute to research on work and family by elucidating what happens on a working from home day, why it has a work‐family conflict‐reducing potential, and how work‐family conflict experiences spill over to the next day. First, we are among the first (see also Delanoeije et al., 2019, and Delanoeije & Verbruggen, 2020) to relate working from home to work‐family conflict on a daily level. However, it remains elusive why precisely working from home has a work‐family conflict‐reducing potential. We examined whether time pressure explains the negative relationship between working from home and work‐family conflict. Consistent with the notion that the very nature of telework supports individuals in saving time (Peters et al., 2004), our results show that on days when individuals work from home, they experience less time pressure than on office days. By focussing on time pressure as a specific work‐related stressor, we went beyond past studies that have merely focused on general stress as an outcome of daily working from home (Delanoeije & Verbruggen, 2020). In line with the resource perspective in work‐family spillover theory, it seems that employees who work from home are left with more resources that can be used to actively participate in the family role, and therefore experience less work‐family conflict. Second, we drew on COR theory (Hobfoll et al., 2018) to posit that experiences of work‐family conflict also extend to the upcoming workday, in terms of employees' energetic levels and how they feel about the organization they work for. Research to date has mostly tested negative spillover effects from work to family within the same day (e.g., Pluut et al., 2018), and little effort has been expended to study overnight effects of work‐family conflict.

The current study suggests that the resource‐draining nature of work‐family conflict has cross‐day implications. Particularly, we found support for the resource loss spiral principle of COR theory, such that experiences of work‐family conflict deplete personal resources (e.g., energy), leaving employees feeling emotionally exhausted and less engaged the next morning. This set of results is in accordance with the process view of the work‐home resources model of Ten Brummelhuis and Bakker (2012), which entails that effects of work‐family conflict develop over time. Moreover, our findings imply that individuals wake up with negative affect about the organization they work for when work has interfered with their family life the previous day. This result provides support for the source attribution perspective of work‐family conflict that proposes that people are likely to become unhappy with the cause of the conflict as they attribute blame to the source (Shockley & Singla, 2011). Our study is among the first to show that the process of source attribution for daily work‐family conflict may translate into negative feelings about the organization individuals work for, which seemingly last till the next workday.

In addition, our study has implications for research on workplace flexibility. Most telework research has been conducted at the between‐person level of analysis (Biron & Van Veldhoven, 2016). Such cross‐sectional studies require employees to place themselves in either a ‘home worker’ or ‘office worker’ category. Although this approach is appropriate when examining differences between the two worker types, it may not portray a realistic picture of how the policy is utilized. In the Netherlands, for instance, approximately one‐fifth (19%) of the working population works from home on an occasional basis (CBS, 2018), indicating that many employees telework on some days and spend the rest of their workdays at the office. Consistent with this trend, there are various calls in the literature (e.g., Allen et al., 2015; Delanoeije & Verbruggen, 2020) to study telework at the within‐individual level because employees' workplace is likely to fluctuate on a day‐to‐day basis. For the current study, we purposefully looked for participants that would show very high within‐person variability for this construct (89.5%, see Table 1). It enabled us to develop an intraindividual model of the daily consequences of telework. Our results show that everyday decisions to work from home or at the office have important implications, not only for how employees experience the workday (i.e., time pressure) and how this affects their home life that day, but also for how they start their next workday. By specifically focussing on the consequences of the utilization of the working from home practice, we believe our study advances research on workplace flexibility.

Finally, we contribute to existing studies that have employed a within‐individual design to study the outcomes of working from home. Thus far, the majority of studies investigating the day‐to‐day consequences of working from home for employees' work‐home interface and work‐related well‐being have focused on the direct relationships between the concepts. For instance, using diary data, Delanoeije and Verbruggen (2020) investigated the influence of working from home on several outcomes, including stress, work‐family conflict, and work engagement. Our aim with the current research was to develop a model that specifies the relationships between concepts that have regularly been related to working from home (i.e., work‐family conflict, work engagement, and affective well‐being) and concepts that have received less attention in this stream of research, such as time pressure and emotional exhaustion. In doing so, we helped to clarify how these variables relate to each other and explain the process by which working from home influences the work‐home interface and ultimately employees' work‐related well‐being.

### 5.2 Practical implications

Our daily diary study holds implications for organizations and employees. First, organizations are highly recommended to offer employees the possibility to work from home, at least on some days, as part of their employment policies. Results from our research revealed that working from home can reduce the likelihood that employees experience conflict between work and family and may aid in shaping more energetic and positive subsequent workdays. We know from research that engaged employees perform better, have more creative ideas, and transfer their energy to co‐workers (Orth & Volmer, 2017; Van Mierlo & Bakker, 2018). Adopting a telework policy may enable employees to successfully manage their work‐home interface and employers can reap the productivity benefits of employees' work engagement.

A second implication is related to our finding that reduced feelings of time pressure (at least partly) account for why working from home results in lower work‐family conflict. It seems that many employees experience the office as a rather stressful work environment that puts them under time pressure, which in turn has negative consequences for their work‐home interface. Organizations need to take proactive measures in regard to this problem. We believe solutions can be found in the domains of social support and stress management. Prior research has shown that daily social support from supervisors can reduce the strain caused by work demands and thus aid in alleviating experiences of work‐family conflict among employees (Pluut et al., 2018). We therefore suggest supervisors to help employees manage their time effectively and find non‐disturbing workspaces to minimize work‐related interruptions. Moreover, in terms of stress management, supervisors can help employees to change their appraisal of time pressure. Although we did not measure challenge and hindrance appraisals of time pressure, our finding that time pressure had unfavourable consequences for individuals' work‐home interface seems to suggest that employees in our sample appraised time pressure as a hindrance. However, there is also research suggesting that daily experiences of time pressure may have a motivating effect and increase workers' engagement (Baethge et al., 2018). It therefore seems worthwhile to think of interventions that may help employees appraise time pressure as a challenge instead of a hindrance in order to reduce its negative effect on individuals' work‐home interface.

Finally, our results have crucial implications for employees. Employees who can make use of the working from home policy need to become aware that their everyday decision to work from home or at the office has critical consequences in terms of well‐being. Our findings suggest that home days are less resource depleting than office days because employees experience less time pressure. Frequent exposure to a work‐related stressor, such as time pressure, may not be sustainable on the long term (e.g., becoming burned out, see Schaufeli & Buunk, 2003). Thus, it is key that individuals seek the opportunity to work from home for at least some portion of the week. In fact, we recommend employees to schedule recurring working from home days in their calendar to ensure that they switch sufficiently between office and home days. That being said, employees need to remain mindful that telework comes with its own set of challenges, which, if not managed properly, might have negative consequences for their work relationships (Golden, Veiga, & Dino, 2008). Teleworkers experience greater levels of professional isolation and lower levels of workplace inclusion than office‐based workers (Morganson et al., 2010). To overcome the physical distance with their colleagues and to reduce feelings of isolation, employees should adopt informal communication methods to keep in contact with their co‐workers on working from home days (Fay, 2011). In addition, employees are recommended to work from home on days when no to little teamwork is scheduled to avoid missing out on developing closer social relationships with their colleagues. No doubt the COVID‐19 pandemic offers many lessons learned in this respect (see e.g., Wang et al., 2020).

### 5.3 Limitations and directions for future research

We should note several limitations of the current research. The first limitation relates to our modest sample size. The data for the current study were collected among 34 employees and 24 spouses. While we acknowledge that our sample size is lower than those used in other daily working from home studies (e.g., Delanoeije et al., 2019), we detected similar significant within‐person (i.e., day) effects. For instance, we noticed that the within‐individual study conducted by Delanoeije and colleagues (2019), using a larger sample size (*n* = 81 and 678 data points), reported an effect size of working from home on work‐family conflict that was comparable to the one found in our study (i.e., *B* = −0.58 and *B* = −0.60, respectively). While this gives us confidence in the results, the magnitude of our effect sizes should be approached with some caution as they might be biased by the small sample size of our study (Gabriel et al., 2019).

Second, we found a remaining direct effect in addition to the indirect relationship between working from home and work‐family conflict mediated by time pressure. The unexplained direct effect implies there may be additional mediators that explain the relationship between our study variables (Zhao et al., 2010). We concur with Rucker and colleagues (2011) that partial mediation could provide more avenues for future research while also strengthening theory development. In that respect there is a silver lining to our partial mediation result because it suggests our theoretical framework was incomplete and we should consider the likelihood of omitted mediators in addition to time pressure. For instance, recent research has drawn on boundary theory to argue that increased work‐to‐home transitions (i.e., addressing home demands during working hours) can help explain why individuals experience less work‐family conflict on days when they work from home (see Delanoeije et al., 2019). We believe it is important for future research to take different theoretical perspectives when studying this topic and explore other mediators that could account for the conflict‐reducing potential of working from home.

Third, while we used daily spouse reports of work‐family conflict, common method bias is a possible limitation since our remaining variables were self‐reported (Siemsen et al., 2010). However, considering that on working from home days employees are not in direct contact with their supervisors and colleagues, it would not be feasible to collect multisource data for the other work‐related constructs in our model (e.g., time pressure). Another limitation relates to the generalizability of our findings because our sample consisted exclusively of office workers. Jobs that cannot be performed by means of information technology may not be suitable for telework (Allen et al., 2015) and thus our findings may not extend to all types of workers. For instance, occupations that require presence at the workplace for personal interaction with customers (e.g., healthcare) may not lend themselves to working from home.

The generalizability of our findings is also influenced by the set‐up and context of the telework arrangement. The data of this study were collected prior to the COVID‐19 pandemic. It stands to reason that the observed relationships in our model, and especially the negative association between working from home and time pressure, are less likely to hold under crisis circumstances that force individuals to work from home on a permanent basis. In our theoretical reasoning, we argued that reduced interruptions are one of the factors that may explain why individuals experience less time pressure on days when they work from home. Yet, considering that during the COVID‐19 crisis the number of online meetings has increased as a means to stay connected with each other (Case, 2020) and many employees juggle the demands of work, childcare, and home schooling under the same roof (Bevan et al., 2020), it raises the question of how the pandemic impacts the degree of control employees have over their working from home day. It emphasizes the need for more research that taps into daily experiences of (flexible) workers.

Another limitation relates to the conceptualization of our predictor and outcome variable. First, employees' use of the telework policy was merely assessed in terms of working from home. That is, we specifically recruited employees who mainly choose their home as the location of the worksite on teleworking days. Thus, our sample does not lend itself to examine differences in the effects of various work environments outside of the office. Perhaps working at a cafe yields different results in terms of time pressure because of increased interruptions. For a more nuanced understanding of the daily consequences of the telework policy, future research should collect data from employees who choose to work from different locations outside the office and examine any differences between these telework locations. Second, our outcome variable referred to work‐family conflict (rated both by the focal participant and the spouse) but we did not test directly family in‐role behaviours. To better understand how family life is affected by experiences of time pressure during the workday, future research may want to supplement our model with measures such as spousal interactions and time spent with the family. Existing research provides some guidance on how to do this (e.g., Ilies, Schwind, Wagner, et al., 2007).

Our understanding of the daily consequences of working from home for time pressure is limited as we did not explore mechanisms that could explain this relationship. In line with the theoretical reasoning of previous research that has investigated the consequences of working from home for employees' day‐level stress (see Delanoeije & Verbruggen, 2020), we argued that individuals experience less time pressure on days when they work from home because they are less likely to be interrupted by colleagues, have more time to focus on complex tasks, and have increased autonomy and control to schedule their working day efficiently. Yet, no study, to the best of our knowledge, has empirically investigated these theoretical pathways. We can offer some preliminary insights as we have data showing that employees in our sample had fewer interruptions during the workday when working from home compared with at the office.^3^ Moreover, our data indicate that the individuals in our sample worked less hours on working from home days than on office days (an average of 6.9 and 8.1 hours, respectively) (cf. Grant et al., 2013). Nevertheless, it would be a fruitful avenue for future research to examine why working from home has a time pressure‐reducing potential and also assess which mechanism in particular is most likely to explain the daily relationship between working from home and time pressure.

Finally, our findings on the next‐day consequences of work‐family conflict raise questions about the potentially persistent nature of strain experienced as a result of time pressure. In order to shed more light on this cross‐day spillover process, it would be valuable to explore mediators that explain why experiences of work‐family conflict negatively affect work‐related well‐being the next morning as well as moderators that explain when or for whom these effects are more likely to hold. As previous research has shown that psychological detachment from work and sleep quality predict negative affect and fatigue the next morning before going to work (Sonnentag et al., 2008), it would be interesting to examine whether evening recovery experiences alleviate the negative effects of work‐family conflict on next morning psychological states. Similarly, lifestyle behaviours may enable individuals to avoid cross‐day spillover and break the vicious circle, as it has been shown that a healthy lifestyle can help maintain well‐being in the face of blurred work‐life boundaries due to working from home (Pluut & Wonders, 2020).

## CONFLICT OF INTEREST

The authors have no conflict of interest to declare.

## ETHICS STATEMENT

All participants involved in the study gave informed consent to participate in the current research. To protect anonymity, participants were requested to come up with identification codes made up by themselves. Participants were informed from the start about their right to withdraw from the study at any point.

## Data Availability

For confidentiality reasons, the original data are not publicly shared.

## ENDNOTES

^1^
In the current paper, the term work‐family conflict refers to work interfering with family.

^2^
As employees' sleep quality and prospect of working from home versus at the office (the next day) might influence their psychological states in the early morning, we controlled for sleep quality (‘How would you rate your sleep quality last night overall?’; 1 = *very bad to* 4 = *very good*) and workplace_t+1_ in the cross‐day relationships between work‐family conflict in the evening and psychological states the next morning (H3–H5b). Results are robust to the inclusion of these control variables. We thank the anonymous reviewers for suggesting these additional analyses.

^3^
Participants responded to the following item: ‘During most of my working day, I could work without being interrupted’ on a scale from 1 = *strongly disagree* to 5 = *strongly disagree*. Results indicate that *M* = 3.1 for office days and *M = *3.8 for working from home days.
